# Effect of suppression of arabinoxylan synthetic genes in wheat endosperm on chain length of arabinoxylan and extract viscosity

**DOI:** 10.1111/pbi.12361

**Published:** 2015-03-27

**Authors:** Jackie Freeman, Alison Lovegrove, Mark David Wilkinson, Luc Saulnier, Peter Robert Shewry, Rowan Andrew Craig Mitchell

**Affiliations:** ^1^ Plant Biology and Crop Science Rothamsted Research Harpenden UK; ^2^ INRA Centre de Recherche Angers‐Nantes Nantes France

**Keywords:** grass xylan, Type II cell wall, wheat flour‐processing properties

## Abstract

Arabinoxylan (AX) is the dominant component within wheat (*Triticum aestivum* L.) endosperm cell walls, accounting for 70% of the polysaccharide. The viscosity of aqueous extracts from wheat grain is a key trait influencing the processing for various end uses, and this is largely determined by the properties of endosperm AX. We have previously shown dramatic effects on endosperm AX in transgenic wheat by down‐regulating either TaGT43_2 or TaGT47_2 genes (orthologues to *IRX9* and *IRX10* in Arabidopsis, respectively) implicated in AX chain extension and the TaXAT1 gene responsible for monosubstitution by 3‐linked arabinose. Here, we use these transgenic lines to investigate the relationship between amounts of AX in soluble and insoluble fractions, the chain‐length distribution of these measured by intrinsic viscosity and the overall effect on extract viscosity. In transgenic lines expressing either the TaGT43_2 or TaGT47_2 RNAi transgenes, the intrinsic viscosities of water‐extractable (WE‐AX) and of a water‐insoluble alkaline‐extracted fraction (AE‐AX) were decreased by between 10% and 50% compared to control lines. In TaXAT1 RNAi lines, there was a 15% decrease in intrinsic viscosity of WE‐AX but no consistent effect on that of AE‐AX. All transgenic lines showed decreases in extract viscosity with larger effects in TaGT43_2 and TaGT47_2 RNAi lines (by up to sixfold) than in TaXAT1 RNAi lines (by twofold). These effects were explained by the decreases in amount and chain length of WE‐AX, with decreases in amount having the greater influence. Extract viscosity from wheat grain can therefore be greatly decreased by suppression of single gene targets.

## Introduction

The cells of the wheat starchy endosperm have primary cell walls which are laid down as the cells expand rapidly to accommodate the deposition of starch and storage proteins. The polysaccharide composition of these walls is unusual, having a low cellulose component (3%), 7% glucomannan, 20% β‐(1,3;1,4) glucan and 70% arabinoxylan (AX) (Mares and Stone, 1973). Pectin and xyloglucan are rarely detected biochemically, but it has recently been shown that there are traces detectable by immunocytochemistry after the removal of AX and β‐(1,3;1,4) glucan (Chateigner‐Boutin *et al*., 2014). The dominant polysaccharide AX contains xylose residues which may be monosubstituted with α‐(1,3) linked arabinofuranose (Araf) or disubstituted with both α‐(1,2) and α‐(1,3) linked Araf. A minority of the α‐(1,3) Araf have ester‐linked ferulic acid (FA) which can oxidatively dimerize to cross‐link AX chains. Compared to the AX or glucuronoarabinoxylan (GAX) in the cell walls of other tissues of grasses, the endosperm AX has a relatively simple structure, with low amounts of FA, lacking glucuronyl substitution and is nonacetylated at maturity. The simple AX structure and low cellulose content of wheat starchy endosperm cell walls may allow them to be readily digested during germination, facilitating access to the reserves (Burton and Fincher, 2014).

The properties of wheat endosperm AX have important consequences for different end uses. It is the main source of dietary fibre in white flour products and, as the soluble fraction of endosperm AX is the most abundant soluble polymer in wheat grain, it largely determines the amount of soluble fibre and also the extract viscosity from whole grain (Gebruers *et al*., 2008). Some of the benefits of soluble fibre in human diet may be related to their properties in affecting viscosity of digesta (Topping, 2007). By contrast, high extract viscosity is a detrimental trait for nonfood uses of wheat, increasing the cost of alcohol production and having a negative impact on growth of monogastric livestock and poultry fed on wheat (Annison, 1991; Yin *et al*., 2004).

The extract viscosity is largely determined by the solubility of AX and the properties of the soluble fraction. Endosperm AX is typically 20–30% water extractable (WE‐AX) and 70–80% water unextractable (WU‐AX). The solubility of AX molecules is affected by the degree of Araf substitution and the distribution pattern of Araf residues on the xylan backbone (Hoije *et al*., 2008; Kohnke *et al*., 2011): Araf substitution hinders hydrogen bonding between xylan chains and favours solubility of the polymer. Cereal grains AX are generally soluble in water at neutral pH, naturally or after alkaline extraction, provided that substitution degree is higher than 30% (Ying *et al*., 2013). Conversely, solubility is decreased by covalent cross‐linking between chains due to FA dimerization. Alkaline extraction removes these ester‐linked FA and increases solubility, and the majority of the wheat endosperm WU‐AX can be solubilized in this way (Gruppen *et al*., 1992). For a given concentration of extracted AX, the viscosity is entirely determined by the chain length of the AX polymer and is unaffected by the degree of Araf substitution (Dervilly‐Pinel *et al*., 2001). The chain length of wheat endosperm AX is in the range of 1000–4000 Xyl residues, compared to around 100 Xyl residues for xylan in Arabidopsis secondary cell walls. Estimates of about 1500–2000 Xyl in typical wheat endosperm WE‐AX chains were obtained from viscometry and laser light scattering measurements (Dervilly‐Pinel *et al*., 2001) and are compatible with reducing end determinations (Courtin *et al*., 2000).

Our knowledge of the genes responsible for synthesis of xylan in general and wheat AX in particular has increased markedly in recent years. Genetic studies in Arabidopsis have identified genes from three main functional groups as being required for normal extension of the xylan backbone; these are the groups containing the Arabidopsis genes *IRX9* and *IRX14* in glycosyl transferase family (GT) 43 and *IRX10* in GT47. Recently, it has been shown that the IRX10 protein can alone extend xylan chains *in vitro* (Jensen *et al*., 2014; Urbanowicz *et al*., 2014), so it is likely that this is the catalytic unit and that IRX9 and IRX14 are accessory proteins which are essential for xylan extension *in planta*. This is supported by evidence from wheat, where a protein complex was isolated which had xylosyl transferase activity and included proteins encoded by orthologues of *IRX14* and *IRX10* (Zeng *et al*., 2010).

A bioinformatics approach which looked for candidate grass genes for AX synthesis and feruloylation, based on greater expression in grasses compared to dicots, predicted the involvement of genes in the clades containing *IRX9*,* IRX14* and *IRX10*, but by far, the greatest bias in expression among GT families was for GT61 genes (Mitchell *et al*., 2007). Rice and wheat genes in the GT61 family have since been shown to add α‐(1,3) linked Araf to xylan in Arabidopsis, which lacks Araf substitution, providing gain‐of‐function evidence that these genes encode xylan Araf transferases. The genes were therefore named XAT1,2,3 (Anders *et al*., 2012). In wheat starchy endosperm, close homologues of *IRX9*,* IRX14* and *IRX10* are all strongly expressed throughout development, as are several GT61 genes (Pellny *et al*., 2012). We have therefore specifically suppressed the expression of the most highly expressed *IRX9* homologue (TaGT43_2), *IRX10* homologue (TaGT47_2) and GT61 gene (TaXAT1) using RNAi constructs driven by the starchy endosperm‐specific HMW1Dx5 promoter. Suppression of TaXAT1 resulted in a 70–80% decrease in the amount of α‐(1,3) linked Araf in AX from mature starchy endosperm (Anders *et al*., 2012), whereas suppression of TaGT43_2 or TAGT47_2 resulted in up to 50% decreases in the amount of AX, but increased Araf substitution (Lovegrove *et al*., 2013). Despite these large changes to the principal component of the endosperm cell wall, these transgenic lines have apparently normal grain weight and germination. Here, we use these transgenic lines to explore the relationships between these genes, AX properties and extract viscosity.

## Results

We selected three independent lines, each carrying RNAi constructs driven by the HMW1Dx5 endosperm‐specific promoter, targeting three AX biosynthetic genes in hexaploid wheat: TaGT43_2 and TaGT47_2 as described in Lovegrove *et al*. (2013) and TaXAT1 as described in Anders *et al*. (2012). These lines all show segregation patterns consistent with a single locus and homozygous T2 or T3 seed plus corresponding null segregant controls from the same generation were used for the experiments reported here. In the case of GT47_2 line 1, some experiments were carried out on GT47_2‐1 and others on GT47_2‐1s which was confirmed as a sister line by Southern blot analysis (Fig. S1). As expected for constructs with an endosperm‐specific promoter, there was no visible effect of the transgenes on plant growth; they also did not significantly affect grain size nor did they have a visible grain phenotype (Fig. S2). The total AX contents in the starchy endosperm (white flour) for the lines used here are summarized in Table 1.

**Table 1:** Total AX content per unit dry weight flour in RNAi wheat lines from homozygous (H) and azygous segregant control (A) samples

Line	mg AX/g flour dwt		
	A	H	H/A %
GT43_2‐3	21.0 ± 0.2	11.6 ± 0.2	55 ± 1%
GT43_2‐5	23.8 ± 1.5	12.4 ± 0.4	52%*
GT43_2‐6	19.8 ± 0.2	16.0 ± 0.6	81 ± 4%
GT47_2‐1	21.6 ± 1.0	11.6 ± 0.7	54 ± 3%
GT47_2‐1s	20.8 ± 0.6	13.9 ± 0.5	67%*
GT47_2‐4	19.5 ± 0.1	12.4 ± 0.3	63 ± 2%
GT47_2‐7	24.0†	16.2†	68%
XAT1‐1	22.0†	19.2†	88%
XAT1‐2	21.0 ± 1.7‡	15.0 ± 0.3‡	73 ± 6%‡
XAT1‐3	21.0 ± 0.6‡	15.1 ± 0.9‡	72 ± 3%‡

Values (mean ± SE) are from 4 independent biological replicates from randomized block design experiment except where otherwise indicated. Measurements are from pentosan assay for GT43_2 and GT47_2, monosaccharide assay for XAT; these assays give directly comparable results (Saulnier *et al*., 1995).

*nonblocked design so no replication of H/A estimate. ^†^no biological replication. ^‡^calculated from previously published data (Anders *et al*., 2012).

### WE‐AX and AE‐AX fractions

The WE‐AX fraction is readily isolated, but we also used an AE‐AX extraction method to allow determination of the properties of WU‐AX by obtaining pure samples representing the majority of the AX in a soluble form. The monosaccharide compositions of the fractions used showed that AE‐AX typically accounted for 80% of the WU‐AX and 60% of the total amount of AX extracted, and xylose (Xyl) plus arabinose (Ara) accounted for ~90% of the sugars in this fraction (Tables S1, S2). The Ara:Xyl ratio was higher in the AE‐AX fraction than in WE‐AX in all samples (Table S3). The Ara:Xyl ratio in WE‐AX was rather variable in azygous samples, and effects of transgenes on this were not consistent; but in AE‐AX, the GT43_2 and GT47_2 RNAi transgenes increased the Ara:Xyl, whereas XAT1 RNAi had little effect.

### AX chain length

**Figure 1:**
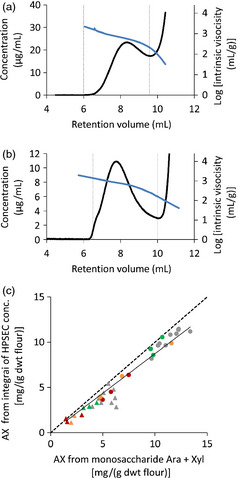
HPSEC analysis of AX extracts from wheat endosperm samples. (a, b) Profiles of concentration (black line) and log intrinsic viscosity (blue line) for WE‐AX (a) and AE‐AX (b) from wild‐type Cadenza flour. Limits for integration calculation are shown (vertical dashed lines); these were maintained the same for all samples. (c) Comparison of integrated concentration from HPSEC profiles with Ara + Xyl content in same samples for GT43_2 RNAi (red symbols), GT47_2 RNAi (orange), XAT1 RNAi (green) and null segregant controls (grey). Triangles are WE‐AX extracts; circles are AE‐AX. Solid line is regression through all points (*y* = 0.87 x; *R* ^2^ = 0.95); dashed line is y = x.

High‐performance size‐exclusion chromatography (HPSEC) was used to examine the size distribution of the AX polymer in these lines using in‐line viscometry to measure the intrinsic viscosity, which is directly related to AX chain length (Dervilly‐Pinel *et al*., 2001). The analyses were carried out on two fractions extracted from the flour samples: a water‐extractable fraction (WE‐AX) and an alkaline‐extractable fraction (AE‐AX) and two extractions of all lines were analysed. Figure 1 shows typical profiles of polysaccharide concentration and intrinsic viscosity against retention time for WE‐AX and AE‐AX fractions; integrated and average values were calculated between retention time limits indicated. Limits were the same for all samples and selected such as to avoid fractions containing significant nonpolysaccharide components; for example, arabinogalactan peptide (AGP) in WE‐AX preparations which elutes at volumes >9.5 mL in Figure 1a. Comparison of integrated AX concentration values against Ara + Xyl content from monosaccharide analysis (corrected for AGP content in WE‐AX samples) shows that the profile between these limits represented ~86% of the AX loaded for both WE‐AX and AE‐AX fractions (Figure 1c). Measured concentrations between the set retention volume limits were decreased to near zero by specific endo‐xylanase treatment, whereas treatment with lichenase, a glucanase specific for β‐(1,3;1,4) glucan, only decreased concentration between the limits by 5% (Fig. S3).

**Figure 2:**
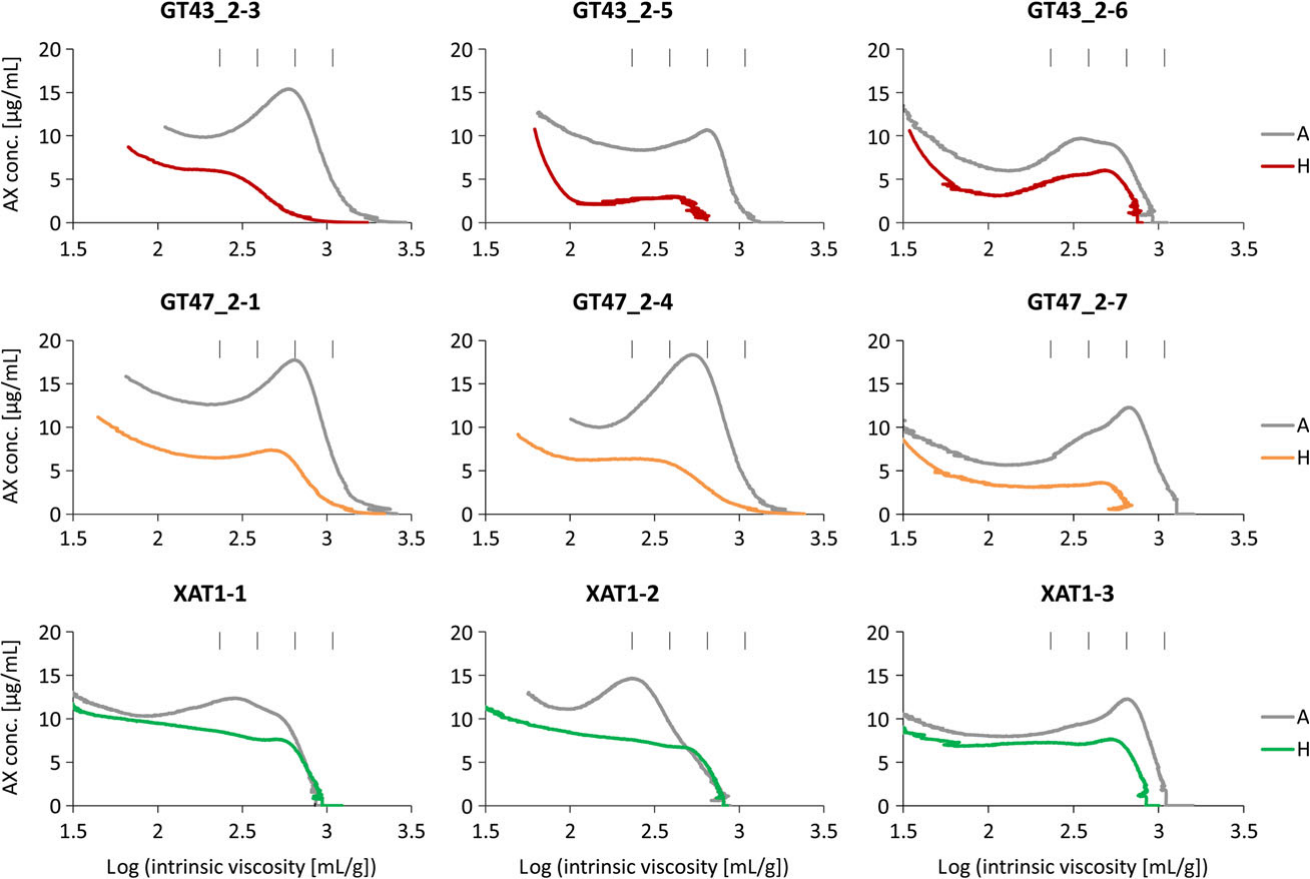
HPSEC profiles of log intrinsic viscosity versus AX concentration for WE‐AX extracts from homozygous transgenic samples (H) and corresponding azygous controls (A). Profiles for GT43_2‐3 and GT47_2‐4 were previously published in a different form in Lovegrove et al. (2013) and are included here for ease of comparison. The four vertical bars indicate expected positions of AX polymers of 500, 1000, 2000, 4000 Xyl length based on relationship in Dervilly‐Pinel et al. (2001).

Figure 2 shows profiles of log intrinsic viscosity against AX concentration for WE‐AX from the transgenic samples and their corresponding controls. We previously reported decreases in the peak and maximum chain length in two GT43_2 lines (GT43_2‐ 3 and GT43_2‐5) (Lovegrove *et al*., 2013), and this was confirmed in a repeat analysis of GT43_2‐5 (Figure 2). However, a third line (GT43_2‐6) did not show these effects, with fairly constant decreases in WE‐AX content over the range of chain lengths. The three lines of GT47_2 RNAi had major decreases in WE‐AX content and did show proportionately greater decreases in longer chain AX, consistent with previously reported results for GT47_2‐4 and a sister line to GT47_2‐1s (Lovegrove *et al*., 2013). The three XAT1 RNAi lines showed decreases in the amount of WE‐AX as previously reported (Anders *et al*., 2012). These lines had not previously been analysed by HPSEC, and the profiles reveal a flatter distribution compared with the controls but that long chains of AX of up to 4000 Xyl still occur.

**Figure 3:**
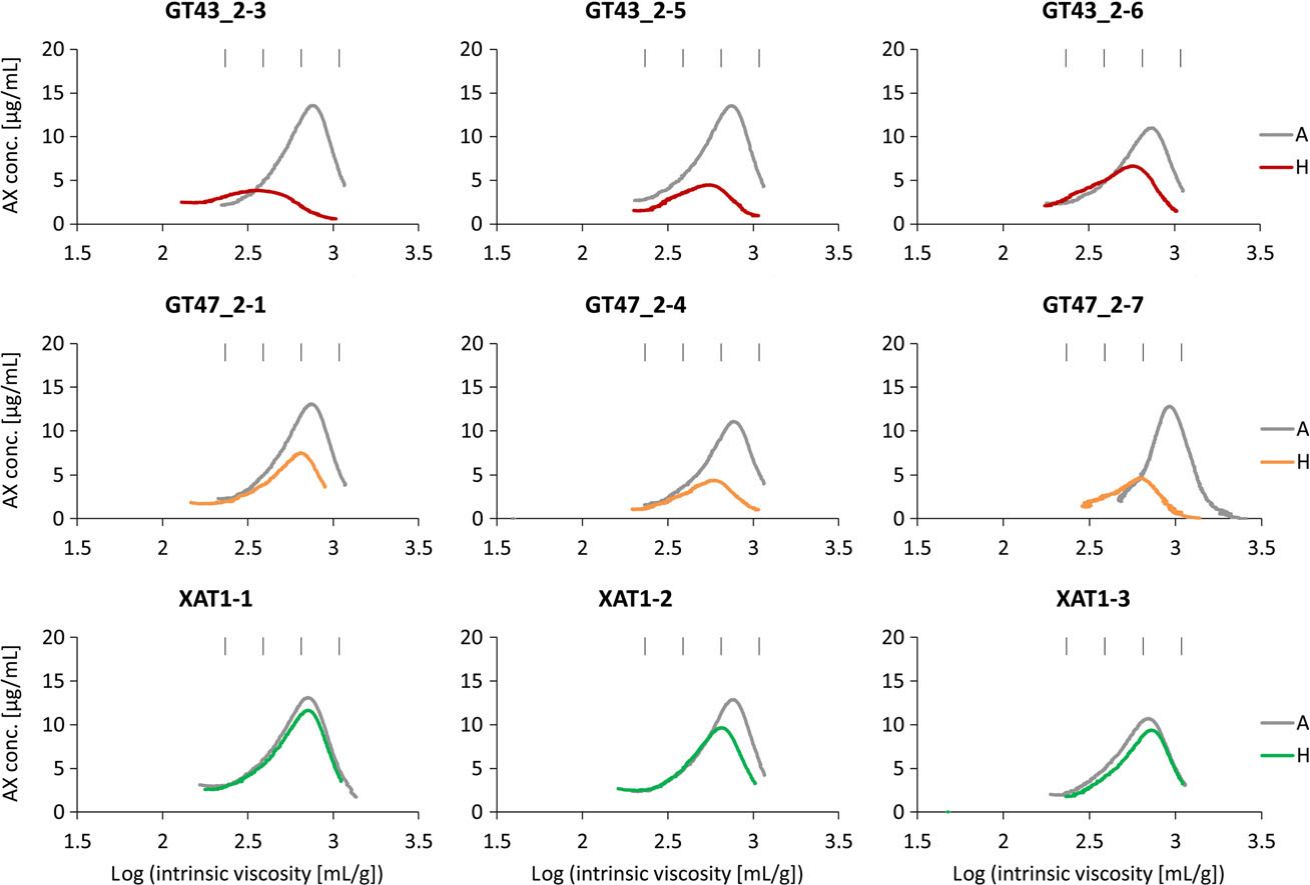
HPSEC profiles of log intrinsic viscosity versus AX concentration for AE‐AX extracts. Details as for Fig. 2.

The profiles of AE‐AX show a more symmetrical size distribution than WE‐AX due to a lack of shorter chain molecules (Figure 3). The AE‐AX profiles from control samples are also more consistent than WE‐AX suggesting that WE‐AX is more sensitive than WU‐AX to environmental factors, as has been previously reported for total WE‐AX content (Andersson *et al*., 1993; Gebruers *et al*., 2010).

Integrated and average values from the HPSEC for both WE‐AX and AE‐AX fractions are summarized in Table 2. The effects on average intrinsic viscosity can be interpreted as effects on AX chain length. For WE‐AX, these are variables between lines for the GT43_2 and GT47_2 constructs, but smaller and more consistent for XAT1 lines. The average chain length of the AE‐AX fraction was decreased in all GT43_2 and GT47_2 lines, but only in one XAT1 line.

**Table 2:** Summary values of AX amount and intrinsic viscosity from HPSEC profiles of WE‐AX and AE‐AX fractions

Sample	WE‐AX						AE‐AX					
	mg/g flour dwt			[η] mL/g			mg/g flour dwt			[η] mL/g		
	A	H	H/A %	A	H	H/A %	A	H	H/A %	A	H	H/A %
Cadenza	4.77			541			9.66			705		
GT43_2‐3	3.38^a^	1.18^a^	35%^a^	550^a^	289^a^	53%^a^	11.18	3.67	33%	692	415	60%
GT43_2‐5	5.42	1.80	33%	366	240	65%	11.47	4.51	39%	681	505	74%
GT43_2‐6	4.77	2.75	58%	328	315	96%	9.65	6.39	66%	667	530	80%
GT47_2‐1	3.94^a^	1.69^a^	43%^a^	556^a^	371^a^	67%^a^	10.94	6.25	57%	698	595	85%
GT47_2‐1s	4.82	1.80	37%	454	271	60%	11.41	6.07	53%	802	630	79%
GT47_2‐4	4.80^a^	1.49^a^	31%^a^	526^a^	469^a^	89%^a^	9.86	3.95	40%	705	502	71%
GT47_2‐7	5.19	2.65	51%	421	198	47%	13.00	5.91	45%	868	538	62%
XAT1‐1	5.78	4.51	78%	309	268	87%	11.27	10.57	94%	680	652	97%
XAT1‐2	5.76	4.40	76%	266	234	88%	10.83	8.59	79%	716	598	84%
XAT1‐3	5.47	3.91	72%	378	293	78%	8.20	9.27	113%	654	645	99%

Amounts are from the integral of concentration profiles, corrected for loadings to express per unit flour. Intrinsic viscosity [η] values are averages calculated as integral of amount x [η] for each point, divided by total amount. Values are average of results from two extractions.

a these values were previously published in Lovegrove *et al*. (2013) and are included here for ease of comparison.

### Relative viscosity

**Figure 4:**
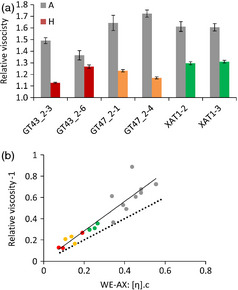
Relative viscosity of water extracts from endosperm samples. As relative viscosity is expressed relative to water, theoretical minimum value is 1. (a) Means ± SE (*n* = 4) grey columns azygous controls, coloured columns homozygous samples. (b) comparison of relative viscosity ‐1 with theoretical relationship predicted from the intrinsic viscosity [η] and amount of WE‐AX estimated for these samples from values in Table 2. Concentration c is given by WE‐AX mg/(g flour) × 0.25 g flour/mL. Symbols are as in Fig. 1C; solid line is *y* = 1.38x + 0.01 from regression, *R* ^2^ = 0.85; dashed line is y = x representing perfect agreement between theoretical and observed.

Intrinsic viscosity is determined by the shape and size of the molecule. AX molecules behave as semiflexible random coils, and variation in intrinsic viscosity reflects variation in chain length but not degree of Araf substitution (Dervilly‐Pinel *et al*., 2001). However, the bulk property of functional importance is relative viscosity (Saulnier *et al*., 1995), the viscosity of aqueous extracts relative to pure water, which depends both on intrinsic viscosity of WE‐AX and its concentration. The relative viscosity of aqueous extracts of replicated flour samples for three lines of each RNAi construct was determined (Figure 4a). Transgenic lines for all three target genes showed significant decreases at *P* < 0.01 relative to their corresponding null segregant controls, except for GT43_2‐6 which had an anomalous low relative viscosity in the control. Relative viscosities were lower for the GT43_2 and GT47_2 RNAi lines than for the XAT1 RNAi lines; the effect in some GT43_2 and GT47_2 RNAi lines decreased the variable portion of relative viscosity (i.e. relative viscosity – 1) by sixfold compared to typical controls, as against twofold for XAT RNAi lines (Figure 4a).

Relative viscosity η_R_ is directly calculable from the intrinsic viscosity [η] and concentration c of the polysaccharide for low concentrations, as

$$\lim_{c\to 0}\eta_{R}-1=\left[\eta \right].c$$

The values from Table 2 for the HPSEC profiles of average intrinsic viscosity and integrated content of WE‐AX samples were used to calculate the theoretical relative viscosity of extracts for 250 mg flour/mL using this equation and are compared with the observed values in Figure 4b. The high correlation between observed and predicted values suggests that the equation holds well at the concentrations of WE‐AX used for determination of relative viscosity. The absolute values are higher for observed relative viscosity than predicted; this is due to the contributions of other components outside the integration limits used on the WE‐AX profiles (Figure 1a) to arrive at the values in Table 2.

The fact that the equation above describes variation in bulk extract viscosity well means that the effect of transgenes on this property can be partitioned between their effects on [η] (due to shorter chain length) and amount of WE‐AX. Thus, the decrease in amount of WE‐AX in GT43_2 and GT47_2 RNAi lines (average −59%) has a bigger effect on η_R_ than the decrease in [η] (−32%); in XAT1 RNAi lines, the decrease in amount (−25%) also has a bigger effect on η_R_ than the decrease in [η] (−16%) although both decreases are of smaller magnitude.

## Discussion

We have previously described the profound effects on AX of suppression of TaGT43_2, TaGT47_2 and TaXAT1 genes by RNAi in wheat endosperm (Anders *et al*., 2012; Lovegrove *et al*., 2013). All three RNAi constructs decreased the total amount of AX; but whereas this was mostly or completely due to a decrease in WE‐AX in XAT1 RNAi lines (Anders *et al*., 2012), the WE‐AX decrease was proportional to the total AX decrease in GT43_2 and GT47_2 RNAi lines (Lovegrove *et al*., 2013). Similar results were obtained here in HPSEC analysis; in that, the total amount of WE‐AX was decreased in all transgenic lines, but the AE‐AX fraction, representing the majority of the WU‐AX, was consistently decreased in GT43_2 and GT47_2 but not in XAT1 RNAi lines (Figures 2 and 3; Table 2).

All three transgenes reduced the average chain length of WE‐AX in all lines (Table 2). In the GT43_2 and GT47_2 RNAi transgenic lines, there was some variation in the effects on chain length between lines and this variation was not obviously related to the magnitude of the effect on the amount of WE‐AX (Figure 2). In some cases, there were severe decreases in the maximum chain length and chain length at peak concentration (lines GT43_2‐3, GT43_2‐5, GT47_2‐1s, GT47_2‐7); but in others, this was less pronounced (GT43_2‐6, GT47_2‐1, GT47_2‐4). These effects were reproducible between experiments on the same line, suggesting that differences in expression of the GT43_2 and GT47_2 RNAi transgenes are likely responsible for the differences in the magnitude of their effects on WE‐AX chain length in different lines with the same transgene. In the XAT1 transgenic lines, there appeared to be little or no effect on maximum chain length, but rather a decrease in the peak concentration (Figure 2), resulting in a decrease in average chain length (Table 2).

The HPSEC profiles for AE‐AX in azygous controls were more consistent than for WE‐AX, indicating that chain length of WE‐AX is more variable than that of AE‐AX and this may be due to greater sensitivity to environment. The effects of transgenes on AE‐AX are also more consistent than for WE‐AX. GT43_2 and GT47_2 RNAi transgenes always decreased the amounts of AE‐AX, ranging from −34% to −67%, and average chain length, ranging from −15% to −40%, with lines with greater effects on amount also tending to have greater effects on chain length (Table 2). The profiles show that chain length at peak concentration was always decreased and maximum chain length was decreased in those profiles where this could be estimated (Figure 3). The XAT1 transgene had little or no effect on the AE‐AX profile except in one line (XAT1‐2) where there was a small effect on amount and chain length (Figure 3).

The effects of the RNAi suppression of GT43_2 and GT47_2 are consistent with the phenotypes of mutants of their respective orthologues in Arabidopsis, *irx9* and *irx10*. Xylan from the secondary cell walls from *irx9* and *irx10* Arabidopsis mutants is decreased in amount and chain length (Brown *et al*., 2009; Pena *et al*., 2007). Here, we have shown for the first time that the same effect occurs in GT43_2 and GT47_2 RNAi wheat lines, not only for WE‐AX but also for the AE‐AX fraction which is representative of the majority of endosperm AX. As WE‐AX and AE‐AX are similarly affected by GT43_2 and GT47_2 suppression, it seems likely that the encoded proteins participate in synthesis of both fractions. There is evidence that the TaGT47_2 protein participates with another GT43‐encoded protein, the orthologue of Arabidopsis *IRX14*, in a xylan synthase complex in wheat (Zeng *et al*., 2010). In Arabidopsis, irx9, irx10 and irx14 mutants have very similar xylan phenotypes (Brown *et al*., 2007, 2009; Pena *et al*., 2007). It is therefore likely that IRX9, IRX10 and IRX14 proteins are components of xylan synthase complexes in dicots and grasses, with IRX10 primarily responsible for catalysis.

The decrease in the amount of WE‐AX previously reported in XAT1 RNAi lines (Anders *et al*., 2012) was confirmed here and is associated with a lowering of the average chain length (Table 2) and a flattening of the profile (Figure 2). The effects on the backbone of AX shown here and by our previous monosaccharide analysis require explanation given that the main effect of XAT1 suppression is a loss of Araf 3‐linked to monosubstituted Xyl (A^3^‐X_mono_). This can be explained if XAT1 protein participates in a complex which synthesizes oligosaccharides containing A^3^‐X_mono_ which are assembled together with other oligosaccharides to make AX chains. In this scenario, when XAT1 is absent, these oligosaccharides would not be made, so the AX chains lack these components. Alternatively, long regions of unsubstituted Xyl in AX resulting from XAT1 suppression could be removed by an endogenous activity after synthesis. In either case, Araf decoration differs in this respect from glucuronic acid decoration in Arabidopsis, which can be removed completely in mutants without affecting xylan chain length (Mortimer *et al*., 2010).

Relative viscosity of aqueous extracts of white wheat flour is largely determined by the WE‐AX from the starchy endosperm, and this is also the case for wholegrain flour as the outer grain layers contribute little to the soluble polymers. Relative viscosity is a detrimental trait for nonfood uses of wheat; it decreases the nutritive value to monogastric animals of wheat animal feed (Annison, 1991; Yin *et al*., 2004), and in alcohol production from wheat grain, it leads to sticky deposits which increase cleaning costs and process downtime. The RNAi suppression of TaGT43_2, TAGT47_2 and XAT1 genes led to decreased extract viscosity (Figure 4a), in the case of TaGT43_2 and TaGT47_2 RNAi of up to 75%. The relative viscosities observed in the transgenic lines (1.1–1.3) are well below the lower limit of the range reported (1.6–4.4) due to natural variation in a diversity screen of 151 wheat varieties (Gebruers *et al*., 2008). The effects of the transgenes can be entirely attributed to the effects on intrinsic viscosity and concentration of WE‐AX (Figure 4b); in TaGT43_2 and TaGT47_2 lines with large decreases in relative viscosity, about 2/3 of the effect was due to decreased concentration, 1/3 due to decreased intrinsic viscosity (Table 2). Therefore, natural or induced loss of function of these genes could improve the suitability of wheat for these nonfood uses. However, as we know that all three homoeologues from the three subgenomes of hexaploid wheat for each of these genes are expressed in starchy endosperm (Lovegrove *et al*., 2013; Pellny *et al*., 2012), this could potentially require the combination of three loss‐of‐function homoeoalleles in a single wheat genotype.

## Experimental procedures

### Plant material and growth conditions

Wheat (*Triticum aestivum* L. var. Cadenza) plants were grown in temperature‐controlled glasshouse rooms as previously described (Nemeth *et al*., 2010).

### Generation of transgenic wheat lines

RNAi construct preparation and wheat transformation were as described in Anders *et al*. (2012) for XAT1 lines and Lovegrove *et al*. (2013) for GT43_2 and GT47_2 lines.

### Quantification of AX content of white flour and white flour extracts

White flour fractions were prepared by milling mature grain as previously described (Anders *et al*., 2012), and the 150 μm fraction (pure starchy endosperm) was used for all analyses. TOT‐AX and WE‐AX contents of flour samples were measured using colorimetric determination of pentosan content as described by Douglas (1981) with modifications as described by Finnie *et al*. (2006). Monosaccharide analysis of samples used for HPSEC (WE‐AX and AE‐AX) and flour and fractions generated for AE‐AX extract method validation was by GLC of alditol acetates. Polysaccharides were hydrolysed in 2 N sulphuric acid (100 °C, 2 h) with inositol as the internal standard, converted to their alditol acetates as described by (Englyst and Cummings, 1984) and run on a Perkin Elmer autosystem XL gas chromatograph with hydrogen as the carrier. AX content was calculated as the sum of arabinose and xylose except in WE‐AX fractions and flour which were corrected for arabinogalactan content assuming an arabinose to galactose ratio of 0.7 (Ordaz‐Ortiz and Saulnier, 2005).

### Sample preparation

WE‐AX for HPSEC analysis was prepared as described in Lovegrove *et al*. (2013). AE‐AX was prepared by a method based on that described by (Gruppen *et al*., 1991). Flour (200 mg) was suspended in 3 mL water containing 100 U/mL thermostable α‐amylase (Megazyme, EC 3.2.1.3) and heated to 95 °C for 15 min. After centrifugation at 1500 *g*, 15 °C for 5 min, the supernatant (=WE in Supplementary Tables S1, S2) containing WE‐AX and glucose (from starch) was removed. The residue was washed in 2 × 3 mL water before suspension in 2 mL saturated barium hydroxide containing sodium borohydride (10 mg/mL) and mixing on a rotating mixer at 25 rpm for 16 h. After centrifugation as above, 1.5 mL of supernatant was removed (=AE‐AX), neutralized to pH5 with 90 μL acetic acid, desalted using a PD minitrap G‐25 column, passed through a 0.45 μm filter and used for HPSEC analysis. For method validation, the residue was washed with 2 × 2 mL water, neutralized to pH5 by addition of 10 μL acetic acid and further extracted in 1.5 mL water with mixing at 25 rpm for 1 h. After centrifugation as above, 1 mL supernatant (=AE2 in Supplementary Tables S1, S2) was removed and the residue (=RES in Supplementary Tables S1, S2) washed with 2 × 1.5 mL water, 2 × 3 mL 95% ethanol followed by 3 mL acetone and dried under infrared lights for approx. 60 min and then under vacuum in the presence of phosphorus pentoxide for 16 h. Samples for relative viscosity measurements were prepared as described by (Saulnier *et al*., 1995) with an additional centrifugation at 10000 *g* before filtration and storage on ice until relative viscosity measurement.

### High‐performance size‐exclusion chromatography

Extracts (50 μL) were injected on the high‐performance size‐exclusion chromatography (HPSEC) system. HPSEC experiments were performed using two slightly different protocols. Protocol 1 used only a Shodex OHPak SB 805 HQ column at 1 mL/min, while Protocol 2 used Shodex OH‐Pak SB 805 HQ and SB 804 HQ columns at 0.7 mL/min. This improved the separation of AX from other compounds and increased retention times, but results were otherwise unchanged. All other method details are exactly as described in Lovegrove *et al*. (2013).

### Relative viscosity measurements

Relative viscosity of aqueous extracts was measured at 30 °C using an automated viscometer (AVS 370, SI Analytics, Germany) fitted with a Micro Ostwald capillary (2 mL, 0.43 mm) and WinVisco software.

## Supporting information


**Figure S1** Southern blot for GT47_2 RNAi lines 1 and 1s. Genomic DNA from plants homozygous for the transgene (1H and 1sH) and their corresponding azygous segregants (1A and 1sA) digested with EcoRI was run in the lanes indicated.
**Figure S2** Grain from transgenic lines and controls.
**Figure S3** HPSEC profiles of AE‐AX samples from azygous wheat lines showing concentration (black lines) and intrinsic viscosity (blue lines) with treatment (dashed lines) or no treatment (solid lines) with recombinant glycosyl hydrolases.
**Table S1** Distribution of monosaccharides between fractions extracted with water and alkali (barium hydroxide) from pure white flour of wheat (*Triticum aestivum* L. var. Cadenza): amount of sugar or arabinoxylan (AX) in each fraction.
**Table S2** Monosaccharide composition of fractions extracted with water and alkali (barium hydroxide) from pure white flour of wheat (*Triticum aestivum* L. var. Cadenza): molar per cent.
**Table S3** A:X ratio of WE‐AX and AE‐AX fractions extracted from white flour of RNAi wheat lines from homozygous (H) and azygous segregant control (A) samples: calculated from monosaccharide analysis with amount of arabinose in WE‐AX samples corrected for AGP content as described in the methods.
