# Supplementary material for: Effect of suppression of arabinoxylan synthetic genes in wheat endosperm on chain length of arabinoxylan and extract viscosity
Source: Plant Biotechnol J. 2015 Mar 27;14(1):109–16. doi: 10.1111/pbi.12361 (PMC5098169; doi:10.1111/pbi.12361)
Supplement: Supplementary file 1 — Figure S1 Southern blot for GT47_2 RNAi lines 1 and 1s. Genomic DNA from plants homozygous for the transgene (1H and 1sH) and their corresponding azygous segregants (1A and 1sA) digested with EcoRI was run in the lanes indicated. Figure S2 Grain from transgenic lines and controls. Figure S3 HPSEC profiles of AE‐AX samples from azygous wheat lines showing concentration (black lines) and intrinsic viscosity (blue lines) with treatment (dashed lines) or no treatment (solid lines) with recombinant glycosyl hydrolases. Table S1 Distribution of monosaccharides between fractions extracted with water and alkali (barium hydroxide) from pure white flour of wheat (Triticum aestivum L. var. Cadenza): amount of sugar or arabinoxylan (AX) in each fraction. Table S2 Monosaccharide composition of fractions extracted with water and alkali (barium hydroxide) from pure white flour of wheat (Triticum aestivum L. var. Cadenza): molar per cent. Table S3 A:X ratio of WE‐AX and AE‐AX fractions extracted from white flour of RNAi wheat lines from homozygous (H) and azygous segregant control (A) samples: calculated from monosaccharide analysis with amount of arabinose in WE‐AX samples corrected for AGP content as described in the methods. [file PBI-14-109-s001.docx]

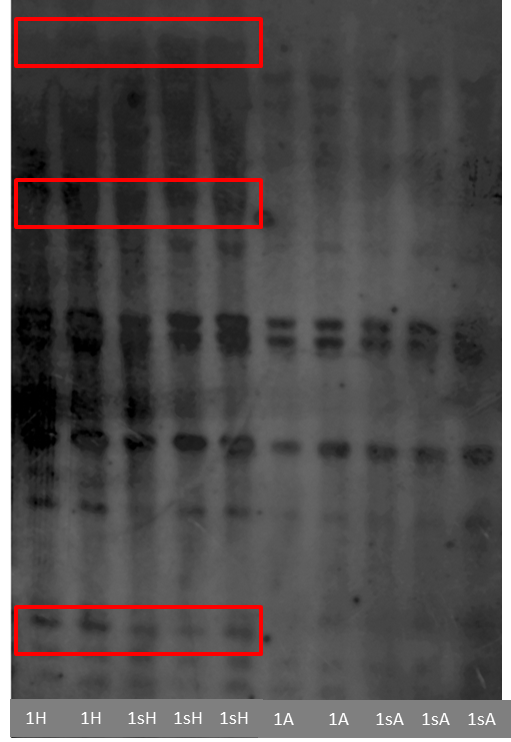


Figure 1S. Southern blot for GT47_2 RNAi lines 1 and 1s. Genomic DNA from plants homozygous for the transgene (1H and 1sH) and their corresponding azygous segregants (1A and 1sA) digested with EcoRI was run in the lanes indicated. Blot was probed with Digoxigenin-labelled probe to the GT47_2 RNAi portion of the transgene; this probe is expected to hybridise with endogenous genes but additional bands present only in transgenics correspond to transgene (in red boxes). Transgenic lines all show the same pattern for these bands indicating that lines 1 and 1s are sisters.


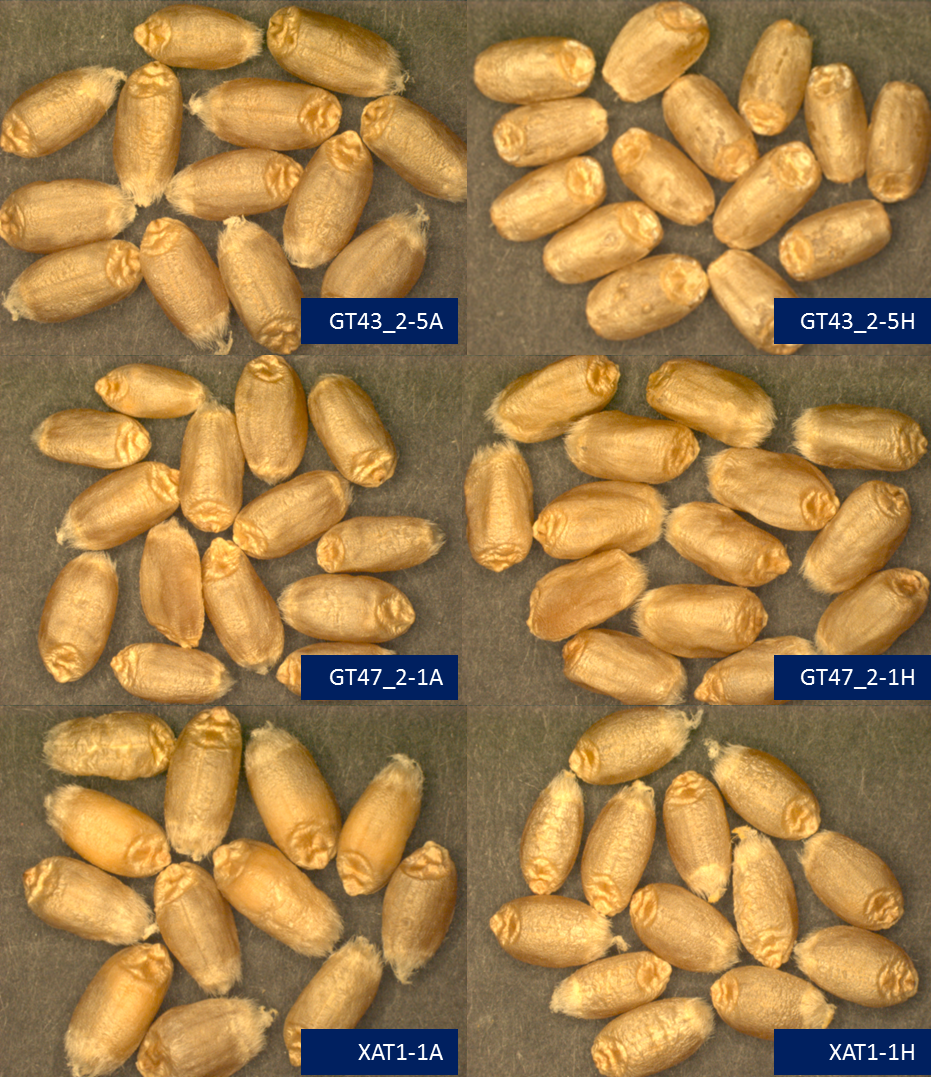


Figure 2S. Grain from transgenic lines and controls.


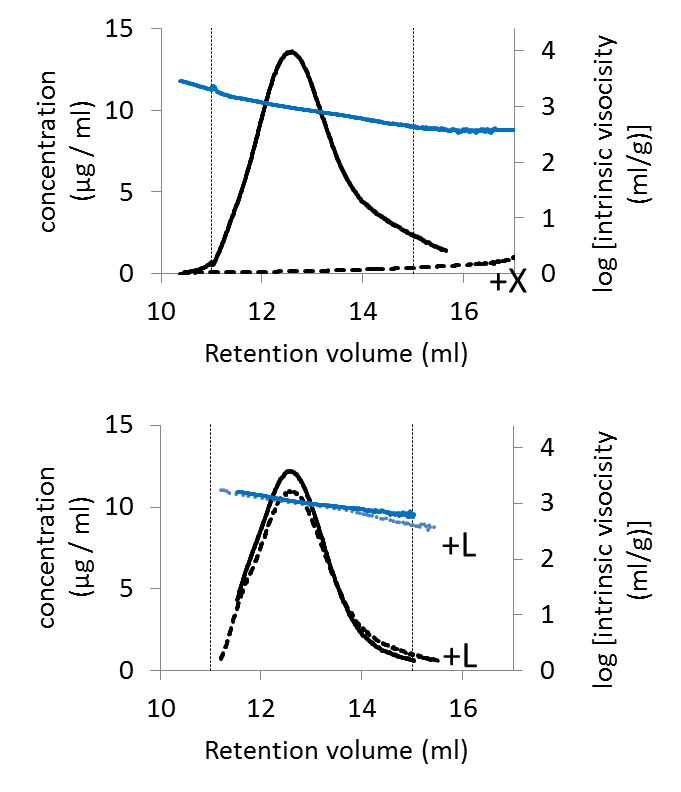


Figure 3S. HPSEC profiles of AE-AX samples from azygous wheat lines showing concentration (black lines) and intrinsic viscosity (blue lines) with treatment (dashed lines) or no treatment (solid lines) with recombinant glycosyl hydrolases. Upper panel: GH11 endoxylanase; no differential viscosity was detected in the presence of the xylanase so this trace is absent. Lower panel: GH16 lichenase. Vertical dashed lines indicate integration limits.

Table S1. Distribution of monosaccharides between fractions extracted with water and alkali (barium hydroxide) from pure white flour of wheat (*Triticum aestivum* L. var. Cadenza): amount of sugar or arabinoxylan (AX) in each fraction. Fractions are from sequential extraction of flour by hot aqueous α-amylase digestion (WE), alkaline (barium hydroxide) extraction (AE-AX), water extraction of the alkaline-treated residue (AE2) and the insoluble residue (RES) (see methods for details). Results are presented for each of two extractions (1 and 2).

|  |  | Monosaccharide content (mg sugar/g dwt flour) | | | | | |
| --- | --- | --- | --- | --- | --- | --- | --- |
| Fraction |  | Arabinose | Xylose | Mannose | Galactose | Glucose | AX |
| Flour | 1 | 11.44 | 16.15 | 3.58 | 3.79 | 849.67 | 26.95 |
|  | 2 | 10.19 | 15.90 | 4.65 | 5.34 | 929.15 | 25.28 |
| WE | 1 | 2.69 | 3.54 | 2.37 | 2.93 | 816.80 | 4.18 |
|  | 2 | 2.42 | 3.27 | 2.01 | 2.31 | 771.60 | 4.08 |
| AE-AX | 1 | 3.65 | 6.56 | 0.33 | 0.39 | 1.16 | 10.21 |
|  | 2 | 3.54 | 6.63 | 0.00 | 0.00 | 1.02 | 10.17 |
| AE2 | 1 | 0.62 | 0.50 | 0.00 | 0.00 | 0.74 | 1.12 |
|  | 2 | 0.52 | 0.42 | 0.00 | 0.00 | 0.86 | 0.94 |
| RES | 1 | 0.69 | 1.06 | 0.08 | 0.05 | 0.82 | 1.76 |
|  | 2 | 0.64 | 0.86 | 0.09 | 0.00 | 0.55 | 1.50 |

Table S2. Monosaccharide composition of fractions extracted with water and alkali (barium hydroxide) from pure white flour of wheat (*Triticum aestivum* L. var. Cadenza): molar percent. Fractions are from sequential extraction of flour by hot aqueous α-amylase digestion (WE), alkaline (barium hydroxide) extraction (AE-AX), water extraction of the alkaline-treated residue (AE2) and the insoluble residue (RES) (see methods for details). Results are presented for each of two extractions (1 and 2).

|  |  | Monosaccharide composition (mol%) | | | | | |
| --- | --- | --- | --- | --- | --- | --- | --- |
| Fraction |  | Arabinose | Xylose | Mannose | Galactose | Glucose | AX |
| Flour | 1 | 1.58 | 2.22 | 0.40 | 0.42 | 95.37 | 3.65 |
|  | 2 | 1.29 | 2.01 | 0.48 | 0.55 | 95.67 | 3.12 |
| WE | 1 | 0.40 | 0.52 | 0.29 | 0.35 | 98.44 | 0.67 |
|  | 2 | 0.38 | 0.51 | 0.26 | 0.29 | 98.56 | 0.69 |
| AE-AX | 1 | 31.06 | 55.87 | 2.30 | 2.71 | 8.06 | 86.93 |
|  | 2 | 32.17 | 60.27 | 0.00 | 0.00 | 7.56 | 92.44 |
| AE2 | 1 | 35.80 | 29.00 | 0.00 | 0.00 | 35.20 | 64.80 |
|  | 2 | 31.91 | 25.52 | 0.00 | 0.00 | 42.57 | 57.43 |
| RES | 1 | 27.43 | 41.97 | 2.53 | 1.70 | 26.38 | 69.40 |
|  | 2 | 31.51 | 42.62 | 3.74 | 0.00 | 22.12 | 74.14 |
| AE-AX average^*^ |  | 32.14 | 59.61 | 0.19 | 1.55 | 6.50 | 91.75 |
| SEM |  | 0.517683 | 0.652654 | 0.19201 | 0.342491 | 0.462815 | 0.755038 |

*Average mol % for all control (azygous and wild-type) samples, n=12

Table S3. A:X ratio of WE-AX and AE-AX fractions extracted from white flour of RNAi wheat lines from homozygous (H) and azygous segregant control (A) samples: calculated from monosaccharide analysis with amount of arabinose in WE-AX samples corrected for AGP content as described in the methods.

|  | A:X ratio | | | |
| --- | --- | --- | --- | --- |
|  | WE-AX | | AE-AX | |
| Line | A | H | A | H |
| GT43_2-3 | 0.37 | 0.29 | 0.50 | 0.70 |
| GT43_2-5 | 0.29 | 0.30 | 0.55 | 0.81 |
| GT43_2-6 | 0.46 | 0.41 | 0.57 | 0.73 |
| GT47-2-1 | 0.38 | 0.56 | 0.52 | 0.76 |
| GT47_2-4 | 0.37 | 0.60 | 0.47 | 0.80 |
| XAT1-1 | 0.31 | 0.41 | 0.52 | 0.56 |
| XAT1-2 | 0.38 | 0.28 | 0.50 | 0.55 |
| XAT1-3 | 0.45 | 0.42 | 0.61 | 0.51 |
| Cadenza | 0.28 |  | 0.56 |  |
